# Morphometric study of the lumbar vertebrae in dried anatomical collections

**DOI:** 10.12688/f1000research.126879.1

**Published:** 2022-11-30

**Authors:** Sharad Ashish, P. Kalluraya, Mangala Pai, B.V. Murlimanju, Y. Rao, Latha Prabhu, Amit Agrawal

**Affiliations:** 1 Department of Anatomy, Kasturba Medical College, Mangalore, Manipal Academy of Higher Education, Manipal, India; 2 Department of Neurosurgery, All India Institute of Medical Sciences, Bhopal, Madhya Pradesh, 462020, India

**Keywords:** Lumbar Vertebrae; Pars Interarticularis; Skeletal Fixation

## Abstract

Background: The objective of this anatomical study was to perform the morphometry of dried lumbar vertebrae in human cadavers.

Methods: This study utilized 200 adult human cadaveric dried lumbar vertebrae. The digital Vernier calipers was used to perform the measurements. The height, antero-posterior length, transverse length of the body of the vertebrae, interpedicular distance at the lateral ends, lamina length, height and thickness, superior and inferior articular facet height and width, mid sagittal and transverse diameter of vertebral foramen, height, width and thickness of the pars inter-articularis were measured.

Results: The vertebral body’s anteroposterior length was more at the lower border than at the superior border ( *p <* 0.01). The length of lamina was higher over the right in comparison to the left (p < 0.001). The height of lamina, width of inferior articular facet, diameter of lateral recess and thickness of pars inter-articularis were greater for the left sided specimens ( *p <* 0.01). The statistical significance was not observed for the comparison of the remaining parameters ( *p >* 0.05).

Conclusion: This anatomical study offered several dimensions of lumbar vertebrae, which are essential in the surgical practice. The implants at the lumbar vertebrae need to be manufactured based on the anatomical dimensions of that particular sample population.

## Introduction

In Latin language, ‘lumbus’ means ‘lion’, hence lumbar vertebrae are compared to a lion. They are very flexible and offer stability to the vertebral column. There are few studies available, which offer the morphometric data of the lumbar vertebrae, however there are not many studies available about the dimensions of pars inter-articularis in the anatomical collections. In the radiographs of the lumbar vertebrae, the pars inter-articularis resembles the neck of a Scottish dog. Since the surgical techniques of the vertebral column involve the utilization of bony anatomical landmarks, the morphometric data of the various parts of the vertebrae are essential. The accurate anatomical dimensional knowledge is important to understand the etiopathogenesis of the lower backache. The bony landmarks like the pars inter-articularis, transverse process, superior and inferior articular facets are particularly important during the internal fixation of the lumbar spine. The pars interarticularis are important parts of lumbar vertebrae, which help during the surgical instrumentation. ^1^ The anatomical studies help in understanding the detail complex morphometry of the vertebral column. ^2^^,^^3^ In this context, the objective of this anatomical study was to perform the morphometry of dried lumbar vertebrae of the human cadavers in sample Indian population.

## Methods

This descriptive anatomical study included 200 adult cadaveric dry lumbar vertebrae. The sample size was calculated by referring the article by Singh *et al..* ^4^

The formula applied was

$$n=2\frac{{\left(Z_{1-\alpha /2}+Z_{1-\beta }\right)}^{2}\sigma^{2}}{d^{2}}$$

Z _1-α/2_= Z value at ‘α’ level of significance

Z _1-β =_ Z value at (1-β) % power

σ= anticipated population standard deviation of the outcome variable (or) common assumed standard deviation between the two groups

d= clinically significant difference

**Figure 1.**
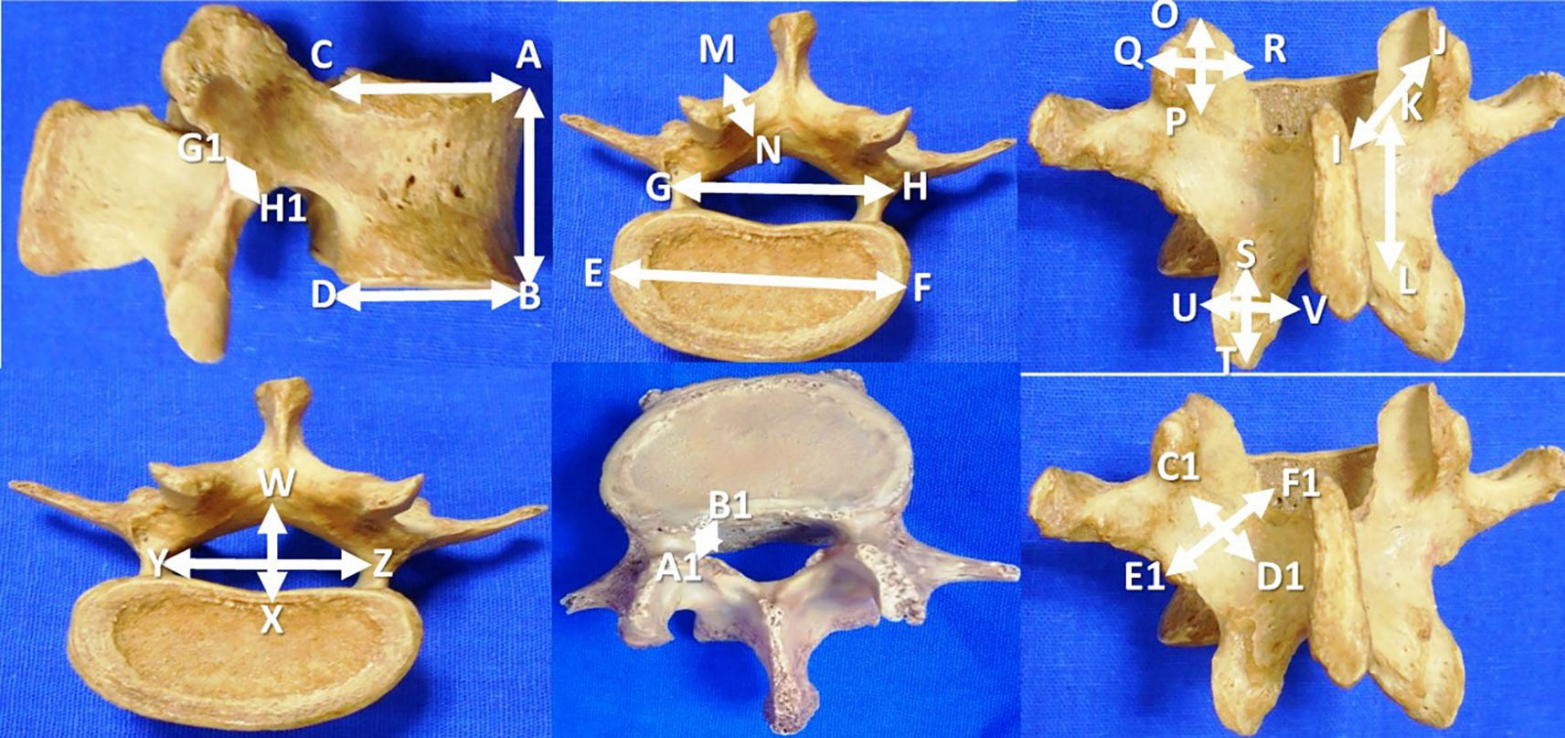
Morphometric parameters of the lumbar vertebrae performed in this study (n=200).

The protocol of this present research is available online at https://www.protocols.io/view/morphometric-study-of-the-lumbar-vertebrae-in-drie-cjqhumt6. The age and gender of the specimens was not taken into consideration. Congenitally deformed lumbar vertebrae were excluded from the present study. Measurements of this study are performed by the digital Vernier calipers. The data are expressed in millimeters and tabulated as mean ± standard deviation. The details of the measurements performed in this investigation are represented in Figure 1 and Table 1. The SPSS software (version 26) was utilized to perform the statistical analysis.

**Table 1.** Morphometric parameters of the lumbar vertebrae (n=200) performed in this study.

1	Vertebral body height (AB)
2	Antero-posterior length of body at superior border (CA)
3	Antero-posterior length of the body at inferior border (DB)
4	Transverse length of the body (EF)
5	Distance between the lateral most parts of the pedicles (GH)
6	Lamina length, right and left side (IJ)
7	Lamina height (KL), right side and left side
8	Lamina thickness (MN) on right and left sides
9	Superior articular facet height (OP) and width (QR), right and left side
10	Inferior articular facet height (ST) and width (UV), right and left side
11	Mid sagittal anteroposterior diameter of vertebral foramen (WX)
12	Transverse diameter of vertebral foramen (YZ)
13	Lateral recess diameter, right and left side (A1B1)
14	Pars inter-articularis height on the right and left sides (C1D1)
15	Pars inter-articularis width on the right and left sides (E1F1)
16	Pars inter-articularis thickness over the right and left sides (G1H1)

The ethics committee of our institution has approved this research (Institutional Ethics Committee, Kasturba Medical College, Mangalore, IEC KMC MLR: 02/2022/60, dated 17.02.2022). Since this is a study from the cadaveric dried bones, the consent from the participants is not applicable. This was waived by our institutional ethics committee. This present research is following the guidelines of the international ethical standards. Since this is a cross sectional study from the dried lumbar vertebrae of the donated cadavers and did not reveal the identity of the body donor, the written informed consent was not taken from the body donor’s family for the use and publication of this research.

## Results

The anatomical data obtained in this study are given in Tables 2 and 3. The vertebral body anteroposterior dimension was more at its lower border than at the upper ( *p <* 0.01). The length of lamina was higher over the right side (p < 0.001). The height of lamina, width of inferior articular facet, diameter of lateral recess and thickness of pars inter-articularis were greater for the left side ( *p <* 0.01). The remaining parameters, which were compared on the right and left sides did not reveal the difference with respect to the statistical significance ( *p >* 0.05).

**Table 2.** Unpaired measurements of the lumbar vertebrae (n=200).

parameter measured	mean ± SD	
vertebral body height	24.6 ± 1.9	
vertebral body AP length at superior border	30.1 ± 3.3	p < 0.01 *
vertebral body AP length at inferior border	30.6 ± 3	
vertebral body transverse length	44 ± 5.1	
distance between lateral walls of pedicle	42.1 ± 8.7	
mid sagittal AP diameter of vertebral foramen	14.2 ± 1.8	
transverse diameter of vertebral foramen	21.6 ± 2.5	

measurements are in mm; paired ‘t’ test; statistical significance

* p<0.01

**Table 3.** Paired dimensions of the lumbar vertebrae (n=200).

measurement	on right side	on left side	‘p’ value
Length of lamina	11.7±2.1	11.3±2.1	p < 0.001 *
Height of lamina	20.8±3.7	21.4±3.7	p < 0.001 *
Thickness of lamina	6.3±1.1	6.4±1.1	p > 0.05
Height of superior articular facet	12.5±2.3	12.7±2.1	p > 0.05
Width of superior articular facet	11.8±1.9	11.7±1.8	p > 0.05
Width of inferior articular facet	11.2±1.9	11.4±1.8	p < 0.05 *
Height of inferior articular facet	13.4±1.9	13.6±2.1	p > 0.05
Diameter of lateral recess	7.3±1.6	7.5±1.5	p < 0.01 *
Height of pars inter-articularis	41.9±3.8	41.7±3.7	p > 0.05
Width of pars inter-articularis	13.4±2.1	13.3±2	p > 0.05
Thickness of pars inter-articularis	8±1.1	8.4±1.2	p < 0.001 *

measurements are in mm; paired ‘t’ test; statistical significance

* p<0.05-significant; p<0.01-moderately significant; p<0.001-highly significant

## Discussion

If the significant part of vertebral body is involved in a disease, there will be neurological deficits and instability of the back. Internal fixation of vertebral column is the best management available for the traumatic spine injury, lumbar canal stenosis, spondylolisthesis and malignant tumors. The internal fixation offers better stabilization and decreases the duration of the morbidity. The spinal surgery is also performed in prolapsed intervertebral disc and conditions like scoliosis. It was reported that, this is among the hardest surgeries to perform as it is prone for the postoperative complications. ^5^ Krag *et al.* ^6^ performed the morphometry of the vertebrae in cadavers, both manually and radiologically. Characterizing the morphology of the spine among populations, would allow personalizing the conditions under which each individual should be exposed. The morphometric data of the vertebrae are not only useful in the field of neurosurgery, but are also essential to the specialties like neurology and orthopedics. Dimensions of the cervical and thoracic spine were already determined in our collections, few years ago. ^7^ This present study was the continuation of this and here we determined the parameters in the lumbar vertebrae. The morphometrical data of various parts of lumbar vertebrae, procured from this study can be considered as the reference data for our study population.

## Implications and limitations

### Implications

There are not many studies being performed about the morphometry of pars inter-articularis. It offers structural support to the vertebral column and considered as the main support. Pars inter-articularis is a dense cortical bone and is exposed in the posterior approaches. There are morphometrical studies, which are performed by using the radiological methods like utilizing the radiographs and computed tomogram scans. ^8^ The vertebral column robusticity increased significantly over the time affecting the dimensions of the vertebral body as well. ^8^ According to Kapoor *et al.* ^9^, the inter-pedicular distance was 18.5 mm at the first lumbar vertebra, 21.5 mm at the lower lumbar vertebrae. Aly and Amin ^10^ reported that the interpedicular distance in the lumbar vertebrae varies from 17 to 43.4 mm and this increases towards inferior region. Nayak *et al.* ^11^ opined that the dimensions of vertebral foramen are higher in the atypical lumbar vertebrae than in the typical. The height of body of vertebrae was 171 cm in males and 158.2 cm in females. ^8^

### Limitations

In the present study, we could not segregate the vertebrae with respect to their number, age and gender. This can be considered as a limitation of this anatomical research. Since it was just a cross sectional anatomical investigation from the dried vertebrae, the specimens from the same cadaver could not be determined as these are random collections. More studies with larger cohort and validated methods of accurate geometric measurements will be helpful in studying this complex anatomy.

## Conclusion

We report the measurements of parts of the vertebrae of the lumbar region in sample Indian population. It is believed that, these data will help the operating neurosurgeons and spine surgeons during the surgeries like laminectomy and decompression. They are also essential in planning the accurate sizes of the plates and screws in the internal fixation. The implants have to be manufactured depending on the anatomical dimensions of that particular sample population.

## Data Availability

Figshare. LUMBAR VERTEBRAE MORPHOMETRIC DATA.xlsx. DOI: https://doi.org/10.6084/m9.figshare.21307917.v1 ^12^ This project contains the following data:
- This descriptive anatomical study included 200 adult cadaveric dry lumbar vertebrae. The sample size was as per the previous study by Singh et. al. The age and gender of the specimens was not taken into consideration. Congenitally deformed lumbar vertebrae were excluded from the present study. Measurements of this study are performed by the digital Vernier calipers. The data are expressed in millimeters and tabulated as mean ± standard deviation. The details of the measurements performed in this investigation are represented in Fig. 1 and Table 1. The SPSS software (version 26) was utilized to perform the statistical analysis. Data are available under the terms of the Creative Commons Attribution 4.0 International license (CC BY 4.0).
