# Supplementary material for: Morphometric study of the lumbar vertebrae in dried anatomical collections
Source: F1000Res. 2023 Dec 28;11:1408. Originally published 2022 Nov 30. [Version 4] doi: 10.12688/f1000research.126879.4 (PMC10660310; doi:10.12688/f1000research.126879.4)
Supplement: Peer review history

# Reviewer report: Reviewer response for version 4

**DOI:** 10.5256/f1000research.160287.r234001

**Published:** 2024-01-19

**Authors:** Ravi Kant Narayan

**Affiliations:** 1 ESIC Medical College & Hospital, Bihar, India

No further comments.

Is the work clearly and accurately presented and does it cite the current literature?

No

If applicable, is the statistical analysis and its interpretation appropriate?

No

Are all the source data underlying the results available to ensure full reproducibility?

Partly

Is the study design appropriate and is the work technically sound?

Partly

Are the conclusions drawn adequately supported by the results?

Partly

Are sufficient details of methods and analysis provided to allow replication by others?

Yes

Reviewer Expertise:

Morphometrical study of bones, Bioinformatics

I confirm that I have read this submission and believe that I have an appropriate level of expertise to confirm that it is of an acceptable scientific standard.

# Reviewer report: Reviewer response for version 3

**DOI:** 10.5256/f1000research.152503.r209286

**Published:** 2023-11-20

**Authors:** Nicholas Bacci

**Affiliations:** 1 University of the Witwatersrand, Johannesburg, South Africa

This paper presents a morphometric investigation of the lumbar vertebrae of the Indian population with relevance and application to spinal orthopedic clinical practice. The authors put forward a well-structured, detailed manuscript of high scientific quality, including a good ethical standard, and a high level of transparency with the protocol, and data files.

 I have some minor comments and suggestions to improve the manuscript:

**Introduction:**

 1. When referring to the shape of the *pars inter-articularis* as resembling the neck of a Scottish dog, please can a clear morphological description be provided instead?

**Methods:**

 1. Please change the reference made to 'gender' to 'sex' instead as this is a biological study, gender is a social denomination and may not always be applicable in the biological context.

 2. This study is commendable for including repeatability considerations as a lot of studies in anatomy do not do that, while it is a core premise of reproducibility. However, inter-observer error cannot be prevented entirely by simply having only one observer take the measurements. It would be better practice to test the repeatability of the measurements in a subset of the sample (usually 10-15% of the overall sample) by asking a secondary observer to measure the subset of the sample and using a statistical test (e.g., Intraclass Correlation also known as ICC). While this is not common practice in anatomy, if the physical lumbar vertebrae are still accessible it would improve the reproducibility of the overall study conducting these measurements and statistically testing them.

 3. While the majority of morphometric studies tend to follow the same procedure the authors do regarding intra-observer error (i.e, measure 3 times and take the average), it would also be better to confirm this statistically by conducting an ICC on the same data (or a subset of them). If the measurements are consistently taken by one observer incorrectly and then averaged to one value it still might not be representative of the 'true' dimensions of the vertebrae. Statistically confirming the agreement between these measurements would strengthen the quality of the paper. If the intra-observer error is high, it might suggest the requirement for better measurement definitions or suggest using different tools for them in the field of vertebral morphometry in general.

 4. In Table 1, please add dashes between the letters of the landmarks referring to Figure 1 (i.e., Vertebral body height (AB) should be: Vertebral body height (A-B)) and clarify in the table these letters refer to Figure 1, as it may be confusing to a reader without that context.

**Discussion:**

 1. The study could benefit from direct comparison to other populations to contextualize the differences between the Indian population and other populations to manufacturers of vertebral implants. An example of a "recent" study from South Africa that may be worth including is:

 Sani, H.Y., 2018. The morphometric description of the thoracic and lumbar vertebral pedicles in European, African, and mixed populations of South Africa. *The Faculty of Health Sciences, University of the Witwatersrand, Johannesburg*. Available at: https://wiredspace.wits.ac.za/server/api/core/bitstreams/bcb279ea-d4d3-4301-ad99-062294c1aa12/content

 However, this is a master dissertation/thesis (grey literature) and not an academic journal publication and may not fit the requirements of the authors or publishing platform for inclusion. As such this suggestion is by no means a requirement.

Is the work clearly and accurately presented and does it cite the current literature?

Yes

If applicable, is the statistical analysis and its interpretation appropriate?

Yes

Are all the source data underlying the results available to ensure full reproducibility?

Yes

Is the study design appropriate and is the work technically sound?

Yes

Are the conclusions drawn adequately supported by the results?

Yes

Are sufficient details of methods and analysis provided to allow replication by others?

Partly

Reviewer Expertise:

Morphological anatomy, biological anthropology, bone histology, forensic anthropology, facial comparison, facial identification.

I confirm that I have read this submission and believe that I have an appropriate level of expertise to confirm that it is of an acceptable scientific standard.

# Response

**Published:** 2023-12-16

**Authors:** Mangala Pai

**Affiliations:** Kasturba Medical College, Mangalore, Manipal Academy of Higher Education, India

We thank the learned reviewer for the time and expert comments. We have revised the manuscript as per the suggestion.

 1. The accurate definition of the pars interarticularis is given in the introduction section of this revised version.

 2. The word 'gender' is replaced by 'sex' in the whole manuscript.

 3.  In this study, repeatability of the measurements by asking a secondary observer was not performed. It would have been better if a subset of the sample was measured by a secondary observer and intraclass correlation was applied statistically. This information suggested by the reviewer is added as the limitation of our study.

 4. If the measurements are consistently taken by one observer incorrectly and then averaged to one value it still might not be representative of the 'true' dimensions of the vertebrae. Statistically confirming the agreement between these measurements would strengthen the quality of the research. If the intra-observer error is high, it might suggest the requirement for better measurement definitions or suggest using different tools for them in the field of vertebral morphometry in general.

 This is also added as the limitation.

 5. The table 1 is revised as per the suggestion,  like AB is revised as A-B

 6. The given dissertation was referred and since it is a very good scientific literature, it is cited in the revised manuscript with respect to the ethnic variations. 

# Reviewer report: Reviewer response for version 3

**DOI:** 10.5256/f1000research.152503.r209281

**Published:** 2023-11-20

**Authors:** Gyanaranjan Nayak

**Affiliations:** 1 Siksha 'O' Anusandhan Deemed to be University, Bhubaneswar, Odisha, India

First of all, I am privileged to review this article. This seems like a well-conducted research. This is approved by the Institution Ethics Committee which is laudable. Despite working on only dried bones, the authors have ventured to get clearance from the said committee. The collection and analysis of the data seem adequate.

1. However, I would like to comment that the segregation of the lumbar vertebrae into typical and atypical groups might have been done by the authors.
2. I may suggest that segregating the vertebrae in the above manner will add more clarity and specificity to this well-conducted research and aptly written article.
3. The authors also highlight that they have not done the above-mentioned segregation. But as for me, the criteria for segregating the vertebrae into typical atypical classes are rather lucid.
4. The criteria can be easily looked up in any standard anatomy text. Apart from this obvious point, the research appears adequate.

Is the work clearly and accurately presented and does it cite the current literature?

Yes

If applicable, is the statistical analysis and its interpretation appropriate?

Yes

Are all the source data underlying the results available to ensure full reproducibility?

Yes

Is the study design appropriate and is the work technically sound?

Partly

Are the conclusions drawn adequately supported by the results?

Yes

Are sufficient details of methods and analysis provided to allow replication by others?

Yes

Reviewer Expertise:

Human Anatomy, Clinical Anthropology, Cadaveric Anatomy, Histology

I confirm that I have read this submission and believe that I have an appropriate level of expertise to confirm that it is of an acceptable scientific standard.

# Response

**Published:** 2023-12-16

**Authors:** Mangala Pai

**Affiliations:** Kasturba Medical College, Mangalore, Manipal Academy of Higher Education, India

We accept the reviewer opinion and unfortunately, this segregation was not performed. This is added in the revised version as the limitation of the present study.

# Reviewer report: Reviewer response for version 3

**DOI:** 10.5256/f1000research.152503.r209288

**Published:** 2023-11-20

**Authors:** Ravi Kant Narayan

**Affiliations:** 1 ESIC Medical College & Hospital, Bihar, India

1. The study is a repetition of numerous previous published literature.
2. There is nothing novel in the manuscript except for the data from the unnamed population.
3. There is a lack of citations for multiple recently published articles on the same topic, such as Priya *et al.* (2023 ^1^).
4. Further, the discussion part is too thin and could have had much more clinically correlated data.

Is the work clearly and accurately presented and does it cite the current literature?

No

If applicable, is the statistical analysis and its interpretation appropriate?

No

Are all the source data underlying the results available to ensure full reproducibility?

Partly

Is the study design appropriate and is the work technically sound?

Partly

Are the conclusions drawn adequately supported by the results?

Partly

Are sufficient details of methods and analysis provided to allow replication by others?

Yes

Reviewer Expertise:

Morphometrical study of bones, Bioinformatics

I confirm that I have read this submission and believe that I have an appropriate level of expertise to confirm that it is of an acceptable scientific standard, however I have significant reservations, as outlined above.

## References

[1] : Analysing lumbar pedicle morphometry observed via traditional and recent modalities. *J Orthop* .2023;43: 10.1016/j.jor.2023.07.022 17-24 10.1016/j.jor.2023.07.022 37555202 PMC10405167

# Response

**Published:** 2023-12-16

**Authors:** Mangala Pai

**Affiliations:** Kasturba Medical College, Mangalore, Manipal Academy of Higher Education, India

We thank the reviewer for the expert opinion.

 As per the suggestion, recently published literature is cited in this revised version.

 Priya A, Narayan RK, Ghosh SK, Sarangi PK. Analysing lumbar pedicle morphometry observed via traditional and recent modalities. J Orthop. 2023 Jul 22;43:17-24.

 The discussion is added with one more paragraph.

 The references are updated.

# Reviewer report: Reviewer response for version 2

**DOI:** 10.5256/f1000research.143713.r162083

**Published:** 2023-02-09

**Authors:** Srinivasa Rao Sirasanagandla

**Affiliations:** 1 Department of Human & Clinical Anatomy, College of Medicine & Health Sciences, Sultan Qaboos University, Muscat, Oman

The authors have responded to all queries raised for version 1 of the manuscript. I approve all the changes made by the authors in version 2.

Is the work clearly and accurately presented and does it cite the current literature?

Partly

If applicable, is the statistical analysis and its interpretation appropriate?

Partly

Are all the source data underlying the results available to ensure full reproducibility?

Yes

Is the study design appropriate and is the work technically sound?

Yes

Are the conclusions drawn adequately supported by the results?

Yes

Are sufficient details of methods and analysis provided to allow replication by others?

Yes

Reviewer Expertise:

Natural products, osteoporosis, cardiovascular research and Bisphenol A associated toxicity and morphological variations.

I confirm that I have read this submission and believe that I have an appropriate level of expertise to confirm that it is of an acceptable scientific standard.

# Reviewer report: Reviewer response for version 1

**DOI:** 10.5256/f1000research.139335.r156968

**Published:** 2022-12-23

**Authors:** Srinivasa Rao Sirasanagandla

**Affiliations:** 1 Department of Human & Clinical Anatomy, College of Medicine & Health Sciences, Sultan Qaboos University, Muscat, Oman

The present study provides the morphometric data of the lumbar vertebrae in dried anatomical collections in the Indian population. The authors presented the manuscript in a detailed manner, along with protocol, and data availability files. However, I have the following comments for the authors to make the article more effective and scientifically sound.

**Introduction**

- Authors should cite some of the recent articles dealing with the morphometry of lumbar vertebrae. 

**Methods**

- It should be stated how many observers were involved in the data collection.
- This section should explain how the authors avoided observational bias.
- The time of the study's conduct needs to be mentioned. 
- If possible, provide more information about the lumbar vertebrae, such as whether they are typical or atypical.
- The statistical test used in the study is missing, though it is mentioned in the data availability file.
- In Tables 2 and 3, the statistical tests used need to be mentioned in the caption. 
- In Table 2, *p* value representation for AP length needs to be double-checked. 

**Discussion**

- The heading "Implications" in this section may not be appropriate as it deals only with the review of the literature.
- Including information about how the current study's findings differ from those of previous studies will make the paper more interesting. 

Is the work clearly and accurately presented and does it cite the current literature?

Partly

If applicable, is the statistical analysis and its interpretation appropriate?

Partly

Are all the source data underlying the results available to ensure full reproducibility?

Yes

Is the study design appropriate and is the work technically sound?

Yes

Are the conclusions drawn adequately supported by the results?

Yes

Are sufficient details of methods and analysis provided to allow replication by others?

Yes

Reviewer Expertise:

Natural products, osteoporosis, cardiovascular research and Bisphenol A associated toxicity and morphological variations.

I confirm that I have read this submission and believe that I have an appropriate level of expertise to confirm that it is of an acceptable scientific standard, however I have significant reservations, as outlined above.

# Response

**Published:** 2023-01-27

**Authors:** Mangala Pai

**Affiliations:** Kasturba Medical College, Mangalore, Manipal Academy of Higher Education, India

**Reviewer comment - Introduction **- Authors should cite some of the recent articles dealing with the morphometry of lumbar vertebrae. 

**Author Reply: **Two more recent references are added in this revised version.

- Julin M, Saukkonen J, Oura P, *et al.*: Association between vertebral dimensions and lumbar modic changes. *Spine (Phila Pa 1976).* 2021; **46**(46): E415–E425. PubMed Abstract | Publisher Full Text
- Kot A, Polak J, Klepinowski T, *et al.*: Morphometric analysis of the lumbar vertebrae and intervertebral discs in relation to abdominal aorta: CT-based study. *Surg Radiol Anat.* 2022; **44**: 431–441. PubMed Abstract | Publisher Full Text

**Reviewer comment -** **Methods: **It should be stated how many observers were involved in the data collection.

**Author Reply: **All the measurements were performed by one among the authors of this manuscript.

**Reviewer comment -** **Methods: **This section should explain how the authors avoided observational bias.

**Author Reply: **The same author performed the measurements to prevent the inter-observer error. Three measurements were performed and the average of it was taken to prevent the intra-observer error.

**Reviewer comment -** **Methods: **The time of the study's conduct needs to be mentioned. 

**Author Reply: **The study duration was 6 months from 16.02.2022 to 16.08.2022.

**Reviewer comment -** **Methods: **If possible, provide more information about the lumbar vertebrae, such as whether they are typical or atypical.

**Author Reply: **The present study did not segregate the vertebrae into typical or atypical, because of the random collections of the dried vertebrae. We could not number the vertebrae with respect to the lumbar region.

**Reviewer comment -** **Methods: **The statistical test used in the study is missing, though it is mentioned in the data availability file.

**Author Reply: **The paired ‘t’ test was applied to compare the parameters between the right and left sides.

**Reviewer comment -** **Methods: **In Tables 2 and 3, the statistical tests used need to be mentioned in the caption. 

**Author Reply: **It was already given in the footnote of the table; now, in this revised version, this is also added in the caption.

**Reviewer comment -** **Methods: **In Table 2, *p* value representation for AP length needs to be double-checked.

**Author Reply: **The AP length was rechecked and it is correct as the dimension was statistically significant and more for the inferior border.

# Response

**Published:** 2023-07-02

**Authors:** Mangala Pai

**Affiliations:** Kasturba Medical College, Mangalore, Manipal Academy of Higher Education, India

In this revised version, we have removed the headings ‘implications’ and ‘limitations’. The sentence about difference of this study from the previous report is added.
